# Fabrication and Characterization of Polyaniline/PVA Humidity Microsensors

**DOI:** 10.3390/s110808143

**Published:** 2011-08-19

**Authors:** Ming-Zhi Yang, Ching-Liang Dai, Wei-Yi Lin

**Affiliations:** Department of Mechanical Engineering, National Chung Hsing University, Taichung, 402, Taiwan; E-Mails: d099061005@mail.nchu.edu.tw (M.-Z.Y.); g9861019@mail.nchu.edu.tw (W.-Y.L.)

**Keywords:** humidity microsensors, polyaniline, polyvinyl alcohol, MEMS

## Abstract

This study presents the fabrication and characterization of a humidity microsensor that consists of interdigitated electrodes and a sensitive film. The area of the humidity microsensor is about 2 mm^2^. The sensitive film is polyaniline doping polyvinyl alcohol (PVA) that is prepared by the sol-gel method, and the film has nanofiber and porous structures that help increase the sensing reaction. The commercial 0.35 μm Complimentary Metal Oxide Semiconductor (CMOS) process is used to fabricate the humidity microsensor. The sensor needs a post-CMOS process to etch the sacrificial layer and to coat the sensitive film on the interdigitated electrodes. The sensor produces a change in resistance as the polyaniline/PVA film absorbs or desorbs vapor. Experimental results show that the sensitivity of the humidity sensor is about 12.6 kΩ/%RH at 25 °C.

## 1. Introduction

Humidity sensors are important devices that can be used to measure and monitor environmental humidity. Recently, various microsensors have been manufactured using microelectromechanical system (MEMS) technology [1–4]. Humidity sensors fabricated by this technology have the benefits of small size, low cost, high performance and easy mass-production [5,6]. For instance, Li *et al*. [7] presented a resistive humidity sensor fabricated by the multi-user MEMS process. Organic conductive polymer—poly(3,4-ethylenedioxythiophene) synthesized by a electrochemical deposition was adopted as the sensitive material of the sensor, and the sensitive material filled a narrow air gap between two nickel electrodes. The resistance of the sensor changed from 37 to 62 Ω as relative humidity increased from 22 to 99.9 %RH at room temperature. A resistive humidity sensor, proposed by Chen *et al*. [8], contained a freestanding cantilever that was a composite structure comprising a layer of platinum deposited on a silicon nitride layer and covered with a polyimide sensing layer. The cantilever generated a deflection as the polyimide sensing layer absorbed vapor, resulting in the platinum layer produced a change in resistance. It was showed experimentally that the resistance of the sensor decreased from 210 to 70 kΩ as relative humidity increased from 40 to 85 %RH at 40 °C. Kang and Wise [9] developed a capacitive humidity microsensor integrated with a polysilicon heater. Polyimide was the sensitive material of the sensor. The polysilicon heater was used to reduce the recovery time after wetting. The humidity sensor contained thousands of polyimide columns, which all connected in parallel by the suspended upper electrode to form a capacitor, and the columns were located on top of lower electrode. The sensor exhibited a sensitivity of 30 fF/%RH. Su *et al*. [10] utilized MEMS and thick-film technologies to fabricate a resistive humidity microsensor. The sensor consisted of a suspended planar membrane bridged to the silicon substrate with two beams. The sensitive material was a composite film of poly-[3-(methacryloylamino)propyl]trimethyl ammonium chloride and SiO_2_ that was coated on the electrodes. The conductivity of the composite film changed upon adsorbing or desorbing. A capacitive humidity microsensor was presented by Kim *et al*. [11], and the sensor was composed of a substrate with a cavity, a bottom electrode, a polyimide sensing layer and a comb-shaped top electrode. In order to enhance the performance of the sensor, the polyimide sensing layer was etched by an O_2_ plasma asher in accordance with the top electrode passivation. The humidity sensor had a sensitivity of 506 fF/%RH. Lee *et al*. [12] made a capacitive humidity microsensor with a micro-bridge structure using front-side etching with XeF_2_ gas. The sensitive film of the sensor was polyimide, and the film locally cured at a temperature over 350 °C for 1 h by the MEMS microhotplate. The sensitivity of the humidity sensor was 0.77 pF/%RH, and its hysteresis was about 0.61 %RH. Sensitivity is an important characteristic for humidity sensors. Polyaniline/PVA is a material that is highly sensitive to water vapor. Thereby, this study employed polyaniline/PVA to develop a humidity microsensor, and its sensitivity exceeds that of Li *et al*. [7] and Chen *et al*. [8].

Fabrication of MEMS devices using the commercial CMOS process is called the CMOS-MEMS technique [13–15]. This technique has a potential to integrate MEMS devices with circuitry on-a-chip. Micro devices manufactured by the CMOS-MEMS technique usually need a post-CMOS process to coat the functional films [16] or to release the suspended structures [17,18]. In this work, the fabrication of a humidity microsensor using the CMOS-MEMS technique was investigated. The sensor in this case required a post-process to coat a sensitive film of polyaniline/PVA. The post-process utilizes a wet etching to etch the sacrificial layer and the polyaniline/PVA is coated on the humidity sensor. The sensor is a resistive type, and it produces a change in resistance when the sensitive film absorbs or desorbs vapor.

## 2. Preparation of the Humidity Sensor

In our humidity sensor, polyaniline/PVA prepared by sol-gel method was adopted as the humidity sensitive material [19,20]. Preparation steps for the polyaniline/PVA included: (1) aniline (C_6_H_5_NH_2_, 0.68 mL) was dissolved in HCl (105 mL) with vigorous stirring to form a homogenous solution; (2) ammonium persulfate (N_2_H_8_S_2_O_8_, 1.67 g) was dissolved in HCl (40 mL) and added to the aniline solution with stirring for 8 h at room temperature; (3) the mixed N_2_H_8_S_2_O_8_/C_6_H_5_NH_2_/HCl solution was aged at room temperature for 120 h, producing a blackish green sediment; (4) PVA (100 mg) was dissolved in dimethyl sulfoxide (DMSO, 20 mL) with stirring at 80 °C until the mixed solution became homogenous; (5) the PVA/DMSO solution was added to the C_6_H_5_NH_2_/N_2_H_8_S_2_O_8_/HCl residue and stirred at room temperature until a homogeneous solution was obtained; (6) the resulting product was filtered, followed by storage in air at 80 °C for 1 h.

**Figure 1.**
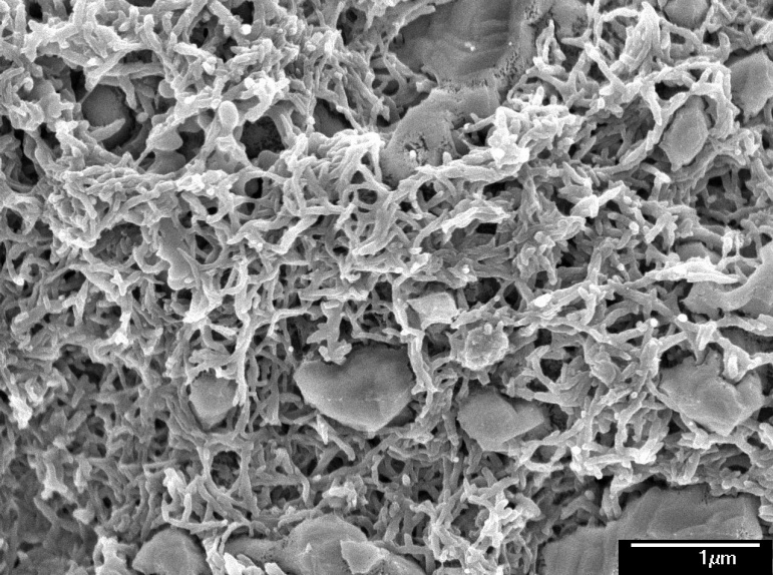
SEM image of the polyaniline/PVA film.

**Figure 2.**
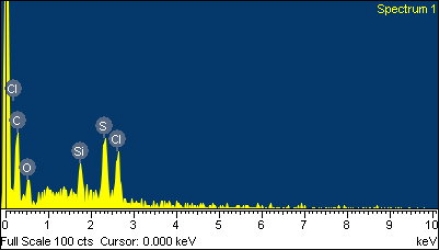
Elements of the polyaniline/PVA film measured by energy dispersive spectrometer.

Scanning electron microscopy (SEM, JEOL JSM-6700F) was employed to measure the surface morphology of the polyaniline/PVA film. Figure 1 presents a scanning electron microscopy image of the polyaniline/PVA film. The sensitive film exhibits micro-porous and nanofiber structures that help increase the sensing reaction because the film has a large surface area. An energy dispersive spectrometer (OXFORD INCA ENERGY 400) was used to detect elements of the polyaniline/PVA. Figure 2 shows the elements of the polyaniline/PVA film measured by energy dispersive spectrometer. Table 1 summarizes composition of the polyaniline/PVA film. The results showed that the polyaniline/PVA film contained 63.68 wt% C, 15.87 wt% O, 8.58 wt% S, 8.06 wt% Cl and 3.80 wt% Si. The Si, Cl and S elements resulted from substrate, DMSO and HCl, respectively.

## 3. Fabrication of the Humidity Sensor

**Figure 3.**
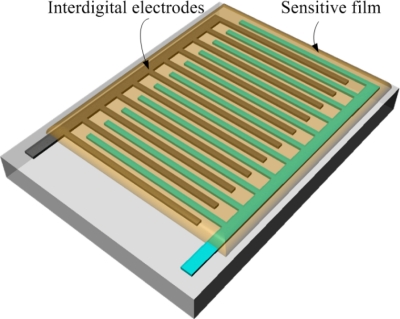
Schematic structure of the humidity sensor.

Figure 3 illustrates the schematic structure of the humidity microsensor. The humidity sensor is constructed of interdigitated electrodes and a sensitive film. The sensitive film of the sensor is polyaniline/PVA, and the film is located on the interdigitated electrodes. The material of the interdigitated electrodes is the stacked metals of the CMOS process. Length, thickness and width of the interdigital electrodes are about 960 μm, 7 μm and 5 μm, respectively, and the gap between the electrodes is 5 μm. The humidity sensor is a resistive type. The resistance variation of the sensor depends on its sensitive film. The humidity sensor generates a change in resistance as the polyaniline/PVA film absorbs or desorbs vapor.

**Figure 4.**
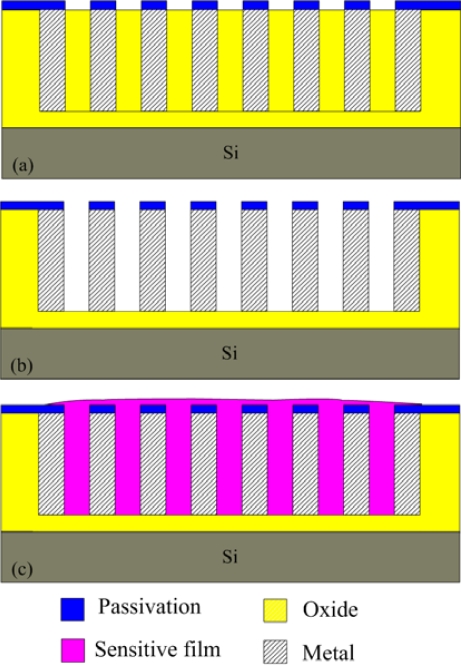
Fabrication flow of the humidity microsensor: (**a**) after the CMOS process, (**b**) etching the sacrificial layer, (**c**) coating the sensitive film.

**Figure 5.**
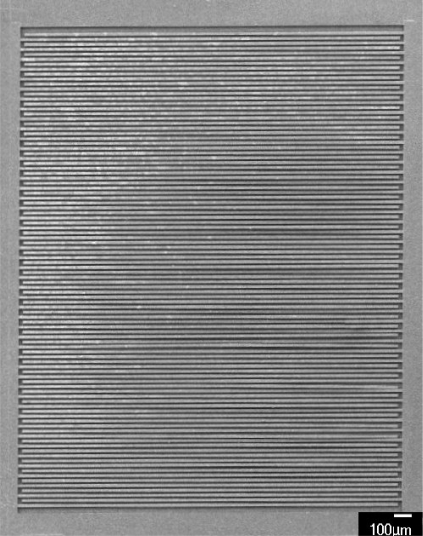
SEM image of the humidity sensor after the wet etching process.

**Figure 6.**
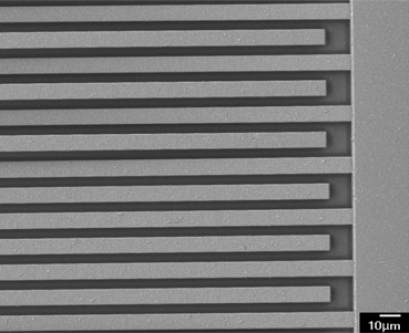
SEM image of the interdigitated electrodes for the humidity sensor.

The humidity microsensor was fabricated using the commercial 0.35 μm CMOS process of Taiwan Semiconductor Manufacturing Company (TSMC). Figure 4 illustrates the fabrication flow of the humidity microsensor. Figure 4(a) shows the humidity microsensor after completion of the CMOS process. The metal layers were used as the interdigitated electrodes. The sacrificial layer was silicon dioxide that was located between the interdigitated electrodes. In order to coat the sensitive film between the electrodes, the sacrificial oxide layer must be removed. The sensor needed a post-process to etch the sacrificial layer and to coat the polyaniline/PVA film. Figure 4(b) displays that the sensor is immersed in Silox to etch the silicon dioxide and to obtain the interdigital electrode gap [21]. Figure 5 shows an SEM image of the humidity microsensor after the wet etching process. Figure 6 presents an SEM image of the interdigitated electrodes for the sensor. Figure 4(c) shows polyaniline/PVA to be dropped using a precision-control micro-dropper. Finally, the polyaniline/PVA was kept in air at 80 °C for 1 h.

## 4. Results and Discussion

A test chamber (GTH-099-40-1P, Giant Force Instruments Enterprise Co.) and an LCR meter were used to measure the performance of the humidity microsensor. The test chamber was able to provide a humidity range of 25–90 %RH and a temperature range of 0–100 °C. Humidity and temperature in the test chamber could be tuned separately and maintained at constant levels. An LCR meter was adopted to measure the resistance of the humidity sensor.

**Figure 7.**
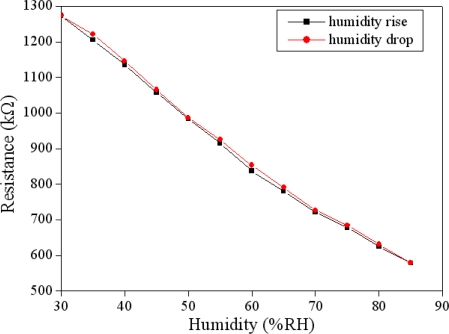
Measured results of the humidity sensor at 25 °C.

The humidity sensor was set in the test chamber. The test chamber provided different humidity to the humidity sensor. The resistance of the humidity sensor changed as humidity in the test chamber rose or dropped. The resistance variation of the sensor was recorded by the LCR meter. Figure 7 shows the measured results of the humidity sensor. In this measurement, the temperature kept constant at 25 °C and the humidity increased from 25 %RH to 85 %RH in 35 min and then dehumidified to 25 %RH at the same rate. The experimental results showed that the humidity sensor almost had no humidity hysteresis. As shown in Figure 7, the resistance of the sensor decreased from 1,285 to 590 kΩ as humidity increased from 25 to 85 %RH, and the sensitivity of the sensor was about 12.6 kΩ/%RH.

**Figure 8.**
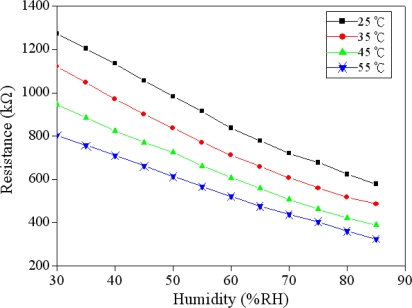
Measured results of the humidity sensor at different temperatures.

**Figure 9.**
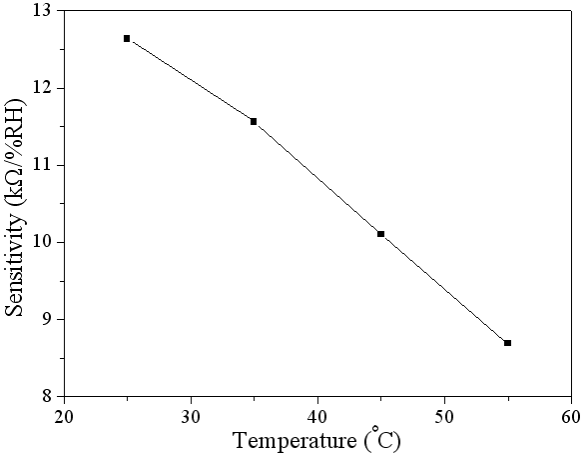
Relation between humidity sensitivity and temperature.

The humidity sensor was tested under different temperatures in order to characterize the influence of temperature by recording the resistance variation of the sensor. Figure 8 shows the measured resistance of the humidity sensor at different temperatures. The curves in Figure 8 are linear in the range of 30–75 %RH. The sensitivity of the sensor can be obtained by the linear fitting to the data in Figure 8. The evaluated results showed that the sensor had a sensitivity of 12.6 kΩ/%RH at 25 °C and a sensitivity of 8.7 kΩ/%RH at 55 °C. In accordance with the results in Figure 8, the relation between the humidity sensitivity and temperature can be obtained, and is shown in Figure 9. The results revealed that the sensitivity of the sensor decreased as the temperature increased, and the sensor had a high sensitivity at room temperature.

Li *et al*. [7] used organic conductive polymer—poly(3,4-ethylenedioxythiophene) to manufacture a resistive humidity microsensor, and its resistance changed from 37 to 62 Ω as relative humidity increased from 22 to 99.9 %RH at room temperature, which the sensitivity of the sensor was about 0.32 Ω/%RH. Chen *et al*. [8] developed a resistive humidity microsensor that the sensitive material was polyimide, and the resistance of the sensor decreased from 210 to 70 kΩ as relative humidity increased from 40 to 85 %RH. The sensor had a sensitivity of about 3.1 kΩ/%RH. This work adopted polyaniline/PVA as a humidity sensitive material, and the sensitivity of the sensor was about 12.6 kΩ/%RH. A comparison with Li *et al*. [7] and Chen *et al*. [8], indicates that the sensitivity of this work exceeds that of Li *et al*. [7] and Chen *et al*. [8].

## 5. Conclusions

A humidity microsensor has been manufactured using the commercial 0.35 μm CMOS process and an appropriate post-process. The humidity sensor had the advantages of small area and high sensitivity. The sensor was composed of interdigitated electrodes and a sensing film. The sensing film was polyaniline/PVA that was synthesized by the sol-gel method, and the film had nanofiber and porous structures that helps to increase the sensor’s sensitivity. The interdigitated electrodes were constructed by the stacked metals of the CMOS process. The post-process used a wet etching to etch the sacrificial oxide layer, and then the polyaniline/PVA was coated on the interdigitated electrodes. The humidity sensor was tested under different temperatures. The results showed that the sensitivity of the sensor decreased as the temperature rose, and the sensor had a high sensitivity (12.6 kΩ/%RH) at room temperature (25 °C).

## Figures and Tables

**Table 1.** Composition of the sensitive film.

**Elements**	**wt%**
C	63.68
O	15.87
S	8.58
Cl	8.06
Si	3.80
